# Recent Changes at Environmental Health Perspectives

**DOI:** 10.1289/ehp.11568

**Published:** 2008-05

**Authors:** Hugh A. Tilson

**Affiliations:** Editor-in-Chief, *EHP*, E-mail: tilsonha@niehs.nih.gov

Readers of *Environmental Health Perspectives* (*EHP*) will note several changes in the masthead of the journal, reflecting a renewed commitment to publishing the best research and news in the environmental health sciences. In the 4 months since I assumed the role of editor-in-chief for *EHP*, I have added 21 new associate editors and 26 new Editorial Review Board members. Associate editors are responsible for managing the peer review of papers and making a recommendation to the journal concerning the acceptability of a manuscript to be published in *EHP*. The Editorial Review Board serves as a pool of potential reviewers for manuscripts submitted to the journal.

I have also established an Advisory Board to provide feedback and recommendations concerning performance and emerging issues facing the journal. This board is made up of 2 NIEHS scientists (Kenneth Korach and Steven Kleeberger), 3 scientists external to the NIEHS (Julian Preston, Russ Hauser, and Philip Iannaccone), and 2 *EHP* editors emeritus (James Burkhart and Thomas Goehl).

After the December 2006 retirement of former acting editor-in-chief James Burkhart, research submissions were screened by a group of interim editors to determine if the manuscript was consistent with the quality, scope, and mission of the journal. This group was led by Kenneth Korach and included NIEHS scientists Matthew Longnecker, Stephanie London, and Michael Waalkes, along with Steven Kleeberger and James Burkhart. In addition to screening papers for subsequent review, this group was responsible for handling the peer review of the remaining papers. *EHP* has been very fortunate to have these dedicated and committed scientists involved in the editorial process over the last year. Today, I head an internal working group that screens all incoming papers. Those papers passing the screening phase are assigned to one of the associate editors for processing.

In 2007, approximately 40% of the 1,108 papers received were deemed worthy of review; the rest were returned to authors without further review. Moreover, the journal averaged approximately 190 pages per issue in 2007, publishing 276 peer-reviewed research articles, 13 full-length reviews, 7 commentaries, and 9 reports of workshops and meetings. The overall acceptance rate for all peer-reviewed papers in 2007 was 19%, down from 29% the previous year. An evaluation of the submission data for 2007 revealed that the average time to first decision for peer-reviewed journals in general was approximately 2 months, whereas the average time from submission to publication in *EHP* was approximately 10 months. However, *EHP* publishes all papers online within 24 hours of acceptance, which averages about 6.5 months from the date of submission.

The masthead also now lists the editors of *EHP* ’s international partnership journals *Mali Médical*, *Ciencia y Trabajo*, *Ciência de Saúde Coletiva*, and the *Journal of Environmental and Occupational Medicine*. As a component of its international program, *EHP* hosts an online version of the quarterly *Mali Médical*, a French-language journal published by the Société de Médecine du Mali. Selected news articles from *EHP* are translated into Spanish for the quarterly *Ciencia y Trabajo*, published by the Fundación Científica y Tecnológica, Asociación Chilena de Seguridad. Abstracts of selected review articles are translated into Portuguese for the quarterly *Ciência de Saúde Coletiva*, published by the Associação Brasileira de Pós-Graduação em Saúde Coletiva. In collaboration with the Shanghai Municipal Center for Disease Control and Prevention (SCDC), more than 30,000 copies of the *EHP Chinese Edition* are distributed in China on a quarterly basis. The SCDC also publishes the *Journal of Environmental and Occupational Medicine*.

Readers will note the presence of the News Committee on the masthead. Formerly identified as “Contributing Editors,” this group helps *EHP* staff identify topics and themes for articles in the Environews section of the journal. Members of the News Committee represent a cross-section of NIEHS scientific and policy leaders knowledgeable about emerging themes and topics in the environmental health sciences. Once a topic is chosen for coverage in the Environews section, *EHP* news staff contact professional science writers to write articles, which undergo subject matter expert review and internal editing. Environews consists of several sections, including the Forum (short reports on current topics of interest); Focus articles (5–8 pages devoted to more in-depth exploration of a wide range of national and international health topics); Spheres of Influence articles (3–4 pages devoted to legal, regulatory, and public health policy issues); Innovations articles (3–4 pages describing new discoveries or approaches in the environmental health sciences); and Science Selections articles (half-page summaries of selected research articles in the concurrent issue). Environews constitutes approximately 10% of each issue.

The overall picture for *EHP* in 2008 is promising. We plan to implement a new web-based manuscript tracking system by late spring of this year. This new system will make it easier to submit and review research papers, and it will allow us to make more effective use of our talented Board of Associate Editors in handling manuscripts. We also have plans to enhance our website and build on existing international partnerships to reach a wider audience outside the United States. Finally, we have received funding to reinstate the *EHP Student Edition*, a free online resource that provides lessons based on *EHP* news articles to high school science teachers. These lessons align with National Science Education Standards in biology, chemistry, environmental science, geology, and physical science. The first new lessons will debut in September 2008. In the meantime, we are conducting a survey to help us direct our efforts to best meet the needs of *EHP Student Edition* users. If you would like to complete the survey, please visit the *EHP* Science Education website (http://www.ehponline.org/science-ed-new/).

## Figures and Tables

**Figure:**
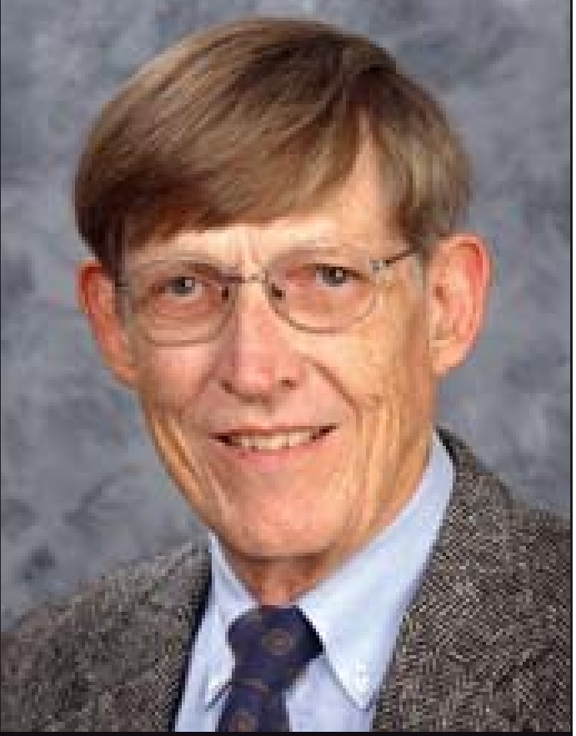
Hugh A. Tilson

